# Comparing impedance cardiography and echocardiography in the assessment of reduced left ventricular systolic function

**DOI:** 10.1186/1756-0500-6-114

**Published:** 2013-03-26

**Authors:** Elzbieta Kaszuba, Sergej Scheel, Håkan Odeberg, Anders Halling

**Affiliations:** 1 Blekinge Competence Centre, Wämö Centre, Karlskrona, SE-371 81, Sweden; 2 Department of Clinical Sciences in Malmö, General Practice/Family Medicine, Lund University, Malmö, SE-205 02, Sweden; 3 Research Unit of General Practice, Institute of Public Health, University of Southern Denmark, Odense C, DK-5000, Denmark

**Keywords:** Heart failure, Reduced left ventricular systolic function, Impedance cardiography, Echocardiography

## Abstract

**Background:**

An early and accurate diagnosis of chronic heart failure is a big challenge for a general practitioner. Assessment of left ventricular function is essential for the diagnosis of heart failure and the prognosis. A gold standard for identifying left ventricular function is echocardiography. Echocardiography requires input from specialized care and has a limited access in Swedish primary health care. Impedance cardiography (ICG) is a noninvasive and low-cost method of examination. The survey technique is simple and ICG measurement can be performed by a general practitioner. ICG has been suggested for assessment of left ventricular function in patients with heart failure. We aimed to study the association between hemodynamic parameters measured by ICG and the value of ejection fraction as a determinant of reduced left ventricular systolic function in echocardiography.

**Methods:**

A non-interventional, observational study conducted in the outpatients heart failure unit. Thirty-six patients with the diagnosis of chronic heart failure were simultaneously examined by echocardiography and ICG. Distribution of categorical variables was presented as numbers. Distribution of continuous variables was presented as a mean and 95% Confidence Interval. Kruskal-Wallis test was used to compare variables and show differences between the groups. A p-value of <0.05 was considered significant.

**Results:**

We found that three ICG parameters: pre-ejection fraction, left ventricular ejection time and systolic time ratio were significantly associated with ejection fraction measured by echocardiography.

**Conclusions:**

The association which we found between EF and ICG parameters was not reported in previous studies. We found no association between EF and ICG parameters which were suggested previously as the determinants of reduced left ventricular systolic function.

The knowledge concerning explanation of hemodynamic parameters measured by ICG that is available nowadays is not sufficient to adopt the method in practice and use it to describe left ventricular systolic dysfunction.

## Background

Chronic heart failure (HF) is a complex syndrome characterized by a long period of sub-clinical symptoms and progressive process associated with poor prognosis. The five-year mortality is six times higher than in general population [1]. The prevalence of chronic HF in general population in Sweden is between 2 and 3% and rises with age to approximately.

10-20% at 70–80 years of age [2,3]. The incidence has increased mainly due to an increasing proportion of the elderly in Sweden [4]. It entails one of the highest costs-of-illness with approximately 2% of the Swedish health care budget [2,5]. Similar data about prevalence, incidence and costs were reported in other European countries [6,7] and USA [8].

The majority of patients at risk of chronic HF, e.g. with coronary heart disease and hypertension, are treated in primary health care.

An early and accurate diagnosis of HF is a big challenge for a general practitioner. Assessment of left ventricular function is essential for the diagnosis of HF and the prognosis. The 5-year survival rate correlates with reduced left ventricular systolic function and decreases in patients with HF to 53% compared with 93% in age- and sex- matched general population [1]. A gold standard for identifying reduced left ventricular function is echocardiography [9]. The European Society of Cardiology considers echocardiography to be mandatory for the establishment of HF diagnosis and highly recommended if HF is suspected [6]. Echocardiography requires input from specialized care. Its accessibility is limited in Swedish primary health care and it is performed only in about 30% of patients with suspected HF [10,11]. Determination of ejection fraction (EF) by echocardiography is routinely used for description of left ventricular systolic function. Potential utility of impedance measurement for assessment of left ventricular function has been suggested since the 1990s [12] with clinical application in patients with HF [13,14]. Impedance cardiography (ICG) is a noninvasive and low-cost method of examination. The survey technique is simple and ICG measurement can be performed by a general practitioner. ICG is considered to be reproducible in ambulatory patients with stable heart failure [15] and has been suggested as a tool to be used by nurses to detect worsening of left ventricular systolic function in patients with heart failure [16].

We aimed to study the association between ICG parameters and the value of EF in echocardiography. If the association was found, ICG could be a method to evaluate reduced left ventricular function.

## Methods

This was a non-interventional, observational study. The study was conducted in the outpatients heart failure unit at the Blekinge County Hospital in Karlshamn in Sweden during the period 6 February 2009 – 6 March 2009. Informed consent was obtained from each participant. There were 63 patients with the diagnosis of chronic HF registered at the heart failure unit. All registered patients were offered participation by a letter send by a cardiologist. No reminder was send. Heart failure unit is the secondary care unit. Patients are referred there from primary care if difficulties with management of chronic HF occur. Diagnosis of chronic HF was established before referral. We thought that it was not necessary to question the diagnosis and we did not penetrate the way it was made.

Exclusion criteria comprised significant aortic valve insufficiency and severe aortic stenosis, both of which influence ICG measurement.

The patients were examined by means of echocardiography and ICG during one consultation.

Both echocardiography and ICG were performed once in each patient. Echocardiography was performed by an experienced cardiologist using Vivid 7, GE equipment. EF was calculated according to modified Simpson’s formula. The following echocardiographic criteria were used to describe left ventricle systolic function: EF ≥ 50% normal systolic function,

EF 40–49% mildly impaired systolic function, EF 30-39% moderately impaired systolic function, EF <30% severely impaired systolic function. ICG was performed by a general practitioner with experience of survey technique using Niccomo™ monitor (Medis. Medizinische Messtechnik GmbH,Germany).

**Figure 1:**
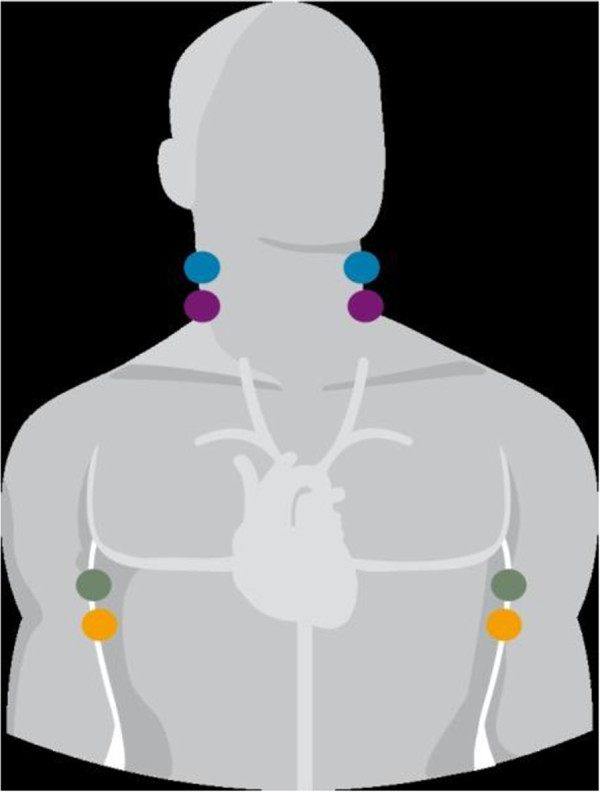
Electrode placement for impedance cardiography measurement.

Two pairs of dual sensors were placed on the patient’s neck and two on the sides of the chest (Figure 1). The skin was prepared in the way similar to the electrocardiography examination. The outer sensors apply a very low constant and alternating current, imperceptible to the patient. The inner sensors measure the baseline impedance of the thorax. Impedance changes with each heartbeat due to changes in the volume and velocity in the aorta. The changes in impedance are used to calculate stroke volume, cardiac output and other hemodynamic parameters. Table 1 contains the list of ICG parameters measured by Niccomo™.

**Table 1:** The list of ICG parameters

**ICG parameter**		**Unit**
Heart rate	HR	1/min
Heart period	HPD	ms
Stroke volume	SV	ml
Stroke index	SI	ml/min
Cardiac output	CO	l/min
Cardiac index	CI	l/min/m^2^
Left cardiac work	LCW	kg · m
Left cardiac work index	LCWI	kg · m/m^2^
Velocity index	VI	1/1000/s
Accelaration index	ACI	1/100/s^2^
Heather index	HI	Ohm/s^2^
Thoracic fluid containce	TFC	1/kOhm
Pre-ejection period	PEP	ms
Left ventricular ejection time	LVET	ms
Systolic time ratio	STR	
Ejection time ratio	ETR	%
Systolic vascular resistance	SVR	ml/min
Systolic vascular resistance index	SVRI	ml/min/m^2^

ICG measurement was obtained after 10 minutes resting, in the supine position with the head elevated between 30–45 degrees for better comfort of the patient. Data were transferred to an external disc and analysed thereafter regarding their adequacy.

None of ICG parameters can be directly compared with EF due to differences in both methods of examinations. ICG parameters were divided into the following groups:

1. Expression for cardiac work: cardiac output, stroke volume, left cardiac work.

2. Contractility: velocity index, acceleration index, Heather index.

3. Fluid status: thoracic fluid content.

4. Expression for systolic function: pre-ejection period, left ventricular ejection time, systolic time ratio and ejection time ratio.

5. Expression for the vascular resistance the heart works against: systolic vascular resistance.

We decided to analyse cardiac output, stroke volume, left cardiac work and systolic vascular resistance related to the body surface as cardiac index, stroke index, left cardiac work index and systolic vascular resistance index.

The study was approved by the research ethics committee at Lund University.

### Statistics

Data were analysed in the STATA version 10 (Stata Corporation, Texas,USA). Distribution of categorical variables was presented as numbers. Distribution of continuous variables was presented as a mean and 95% Confidence Interval. Kruskal-Wallis test was used to compare variables and show differences between the groups. A p-value of <0.05 was considered significant.

## Results

We obtained consent for participation from 37 patients out of 63 patients registered at heart failure unit. Those 37 patients were enrolled into the study. ICG could not be performed in one patient due to distortions in the ICG signal and this patient was excluded from further calculations. The mean age of 36 patients was 68.3 years (CI 64.2-72.4).

The group comprised 28 men (78%) and 8 women (22%). The mean age for men was 68.2 (95% CI 64.0 -72.4) and for women 68.5 (95% CI 54.7-82.3). None of the patients had significant aortic valve insufficiency or severe aortic stenosis. None of the patients reported any discomfort or adverse reaction associated with ICG measurement.

Normal left ventricular systolic function was presented in 13 out of 36 patients (36%), mildly impaired in 9 patients (25%), moderately and severely impaired each in 7 patients, respectively (19%). Three ICG parameters: pre-ejection fraction, left ventricular ejection time and systolic time ratio were associated with EF with significant p-value.

The results are presented in Table 2.

**Table 2:** Distribution of ICG parameters (value and 95% CI) in groups with different ejection fraction

**EF**	**≥50% (n = 13)**	**40-49% (n = 10)**	**30-39% (n = 6)**	**<30% (n = 7)**	**p-value**
CI	2.808 (2.56–3.5)	3.14 (2.42–3.53)	2.6 (2.46–3.29)	2.7 (2.05–2.92)	0.1180
SI	42.15 (37.04–47.27)	35.95 (26.96–44.83)	36.86 (28.77–44.94	38.83 (27.46–50.2)	0.2708
PEP	110.31 (94.38–126,23)	108.4 (95.71–121.1)	139.5 (110.23–168.77)	148 (125.2–170.79)	0.0069*
LVET	323.08 (285.77–360.38)	292.7 (271.06–314.34)	262 (222.78–301.22)	268.43 (212.7–324.15)	0.0462*
STR	0.35 (0.29–0.41)	0.38 (0.32–0.43)	0.54 (0.41–0.66)	0.75 (0.4–0.77)	0.0031*
ETR	37.15 (34.65–39.66)	37.3 (32.02–42.58)	32.66 (30.04–35.29)	34.57 (32.51–36.63)	0.1934
VI	33.77 (29.28–38.26)	38.4 (29.9–46.9)	35.17 (20.42–49.9)	40 (26.12–53.88)	0.61
ACI	51.84 (42.92–60.77)	64.6 (44.59–84.61)	66 (41.33–90.67)	68.14 (50.3–85.98)	0.26
HI	7.9 (5.81–9.98)	10.03 (6.44–13.61)	6.18 (2.98–9.39)	5.8 (2.59–9.0)	0.1006
LCWI	3.17 (2.7–3.63)	3.95 (2.96–4.93)	3.02 (2.5–3.52)	2.87 (2.38–3.35)	0.2624
SVRI	2452 (2099–2806)	2435 (1899–2971)	2662 (2179–3145)	2511 (1743–3279)	0.8549
TFC	35.12 (2.43–3.17)	33.89 (2.65–3.63)	39.63 (2.17–3.02)	43.99 (2.11–3.28)	0.1461

* p value of <0,05.

## Discussion

The purpose of this study was to find whether there is any association between EF measured by echocardiography and hemodynamic parameters measured by ICG.

We found associations between EF and three of four ICG parameters which describe systolic function of the left ventricle: pre-ejection period, left ventricular ejection time and systolic time ratio. We did not find any association between EF and the fourth parameter which describes systolic function - ejection time ratio, though it is directly proportional to left ventricular ejection time. We cannot explain this. The technique of ICG examination was correct. Only one examination had insufficient quality, most likely due to a bad contact between a sensor and the skin. There was no other potential cause of the distortions in ICG signals. We do not think that the patients’ condition could influence the quality of ICG examination and therefore our results. All grades of HF were represented in our study population. None of the patients had unstable HF - a condition which can influence the quality of ICG examination.

A limitation of our study is a small number of patients. Nevertheless, most of the previous studies concerning the correlation between ICG and echocardiography had a small number of participants. This might be a reason why the results are not ambiguous. Evaluation of left ventricular function was the subject in previous studies with ICG but none of the parameters we found associated with EF was previously regarded as a determinant of left ventricular systolic dysfunction. Systolic time ratio, however, was able to distinguish preserved from impaired EF [17]. Also, a close correlation between systolic time ratio and changes in EF was observed before and after treatment in patients with heart failure [18]. An association between systolic time ratio and EF was found in our study.

The following ICG parameters were suggested in another study as determinants of left ventricular systolic dysfunction: cardiac index, left cardiac work index and systolic vascular resistance index [19]. We could not confirm this.

The lack of association between cardiac index and EF found in our study confirms a lack of agreement between absolute values of cardiac output measured by ICG and by echocardiography [20]. We did not find any data about pre-ejection period or left ventricular ejection time as determinants of reduced left ventricular function.

According to the theoretical equation EF is a quotient of stroke volume and end diastolic volume, cardiac output is a product of stroke volume and heart rate. ICG was considered to be a reliable method to determine stroke volume [21]. Owing to these facts, we hypothesized that stroke index and cardiac index would be determinants of left ventricular systolic function and would be associated with EF. ICG was a validated method for measuring cardiac output [22]. Cardiac output by ICG was significantly correlated with a thermodilution method as a gold standard even in patients with heart failure [23,24], which strengthened our hypothesis.

No association between stroke volume index or cardiac output index and EF was found in our study.

EF is commonly used to describe cardiac contractility [25]. We expected to find an association between EF and ICG parameters which describe contractility: velocity index, acceleration index and Heather index. Heather index by ICG has been suggested as the determinant of reduced left ventricle systolic function [26]. No association between EF and those parameters was found in our study.

## Conclusions

The possibility to determine left ventricular function by ICG makes the method attractive for use in patients with HF in primary health care. The association which we found between EF and ICG parameters was not reported in previous studies.

We found no association between EF and ICG parameters which were suggested previously as the determinants of reduced left ventricular systolic function. We do not think that knowledge concerning explanation of hemodynamic parameters measured by ICG that is available nowadays is sufficient to adopt the method in practice and use it to describe reduced left ventricular function.

## Abbreviations

HF: Heart failure; EF: Ejection fraction; ICG: Impedance cardiography

## Competing interests

The authors declare that they have no competing interests.

## Authors’ contributions

EK participated in the design of the study, carried out the data collection, helped with data interpretetion and drafted the manuscript.SS helped with the design of the study, data collection and draft of the manuscript. HO helped draft the manuscript. AH designed the study, performed the statistical analysis, handled the data set, interpreted the data, partcipated in drafting the manuscript. All the authors read and approved the final manuscript.
